# Assessing sleep health in a European population: Results of the Catalan Health Survey 2015

**DOI:** 10.1371/journal.pone.0194495

**Published:** 2018-04-18

**Authors:** Mireia Dalmases, Ivan D. Benítez, Anna Mas, Oriol Garcia-Codina, Antonia Medina-Bustos, Joan Escarrabill, Esteve Saltó, Daniel J. Buysse, Núria Roure, Manuel Sánchez-de-la-Torre, Montse Rué, Ferran Barbé, Jordi de Batlle

**Affiliations:** 1 Group of Translational Research in Respiratory Medicine, Hospital Universitari Arnau de Vilanova and Santa Maria, IRB Lleida, Lleida, Spain; 2 Centro de Investigación Biomédica en Red de Enfermedades Respiratorias (CIBERES), Madrid, Spain; 3 Subdirecció General de Planificació Sanitària i Professional, Catalan Health Department, Barcelona, Spain; 4 Master Plan for Respiratory Diseases (PDMAR), Catalan Health Department, Barcelona, Spain; 5 Center for Sleep and Circadian Science, University of Pittsburgh, Pittsburgh, PA, United States of America; 6 Basic Medical Sciences Department, University of Lleida-IRBLLEIDA, Lleida, Spain; University of Rome Tor Vergata, ITALY

## Abstract

**Objective:**

To describe the overall sleep health of the Catalan population using data from the 2015 Catalan Health Survey and to compare the performance of two sleep health indicators: sleep duration and a 5-dimension sleep scale (SATED).

**Methods:**

Multistage probability sampling representative of the non-institutionalized population aged 15 or more years, stratified by age, gender and municipality size, was used, excluding nightshift-workers. A total of 4385 surveys were included in the analyses. Associations between sleep health and the number of reported chronic diseases were assessed using non-parametric smoothed splines. Differences in the predictive ability of age-adjusted logistic regression models of self-rated health status were assessed. Multinomial logistic regression models were used to assess SATED determinants.

**Results:**

Overall mean (SD) sleep duration was 7.18 (1.16) hours; and SATED score 7.91 (2.17) (range 0–10), lower (worse) scores were associated with increasing age and female sex. Alertness and efficiency were the most frequently impaired dimensions across age groups. SATED performed better than sleep duration when assessing self-rated health status (area under the curve = 0.856 vs. 0.798; p-value <0.001), and had a linear relationship with the number of reported chronic diseases, while the sleep duration relationship was u-shaped.

**Conclusions:**

Sleep health in Catalonia is associated with age and gender. SATED has some advantaged compared to sleep duration assessment, as it relates linearly to health indicators, has a stronger association with self-rated health status, and provides a more comprehensive assessment of sleep health. Therefore, the inclusion of multi-dimensional sleep health assessment tools in national surveys should be considered.

## Introduction

Research in sleep medicine has classically focused on sleep disorders, rather than on the study and promotion of sleep health. Nevertheless, interest in sleep health, understood not only as the absence of a disorder but also as a positive attribute, has recently been growing. In fact, over the past few years, numerous studies have evaluated specific sleep characteristics in relation to the occurrence of chronic diseases and overall health. Short sleep duration has specifically been associated with obesity [1], diabetes [2], hypertension [3,4], coronary heart diseases [5,6], cerebrovascular diseases [7], cancer [7,8] and even all-cause mortality [9]. In addition, long sleep duration has been associated with adverse health outcomes [2,9,10]. There are increasing number of studies examining other sleep dimensions, such as timing or quality. Changes in sleep timing, due to alternating shift work, have been related to hypertension progression [11] and diabetes onset [12]. Finally, poor subjective sleep quality has been associated with worse quality of life [13] and increased carotid atherosclerosis [14], and self-reported disturbed sleep has been suggested to be an independent risk factor for cardiovascular mortality in women [15].

The growing appreciation for the importance of adequate sleep in health maintenance and disease prevention has led to the need for new tools for the measurement of sleep health and all its dimensions. One of the authors (DJB) proposed a self-reported scale including five positive dimensions of sleep/wakefulness–Satisfaction, Alertness, Timing, Efficiency and Duration (SATED)–which can be used to produce a single score ranging from 0 (poor sleep health) to 10 (good sleep health) [16]. Although the SATED scale has not been psychometrically validated, its dimensions are derived from published literature linking sleep characteristics to adverse health outcomes [16]: (i) Satisfaction (the subjective assessment of “good” or “poor” sleep): mortality, metabolic syndrome, diabetes/impaired glucose metabolism, hypertension, coronary heart disease, and depression; (ii) Alertness (the ability to maintain attentive wakefulness): mortality, coronary heart disease, and impaired neurobehavioral performance; (iii) Timing (the placement of sleep within the 24-hour day): mortality, coronary heart disease, metabolic syndrome, diabetes/impaired glucose metabolism, and accidents; (iv) Efficiency (the ease of falling asleep and returning to sleep): mortality, metabolic syndrome, diabetes/impaired glucose metabolism, hypertension, coronary heart disease, and depression; and, (v) Duration (the total amount of sleep obtained per 24 hours): mortality, obesity, metabolic syndrome, diabetes, hypertension, coronary heart disease, and impaired neurobehavioral performance.

The Catalan health authorities in Spain were concerned about the sleep health of the population and thus decided to include several sleep-related questions in the Catalan Health Survey (ESCA) [17]. The ESCA aims to evaluate the health status and health services utilization of the Catalan adult population and previously included a question on sleep duration. In 2015, the SATED scale was translated into Catalan and incorporated into the survey.

The current study analyzed data from ESCA 2015 and aimed to (i) describe the overall sleep health of the Catalan population aged 15 or more years, (ii) assess and compare the ability of the SATED score and a single question on sleep duration to predict self-rated health status, (iii) assess the relative weight of the SATED dimensions in relation to perceived overall health in different age groups, and (iv) evaluate the determinants of the SATED score and the demographic and clinical factors associated with better or worse sleep health.

## Methods

### Design, population and data collection

This study was an observational cross-sectional study based on the ESCA 2015 survey [17]. The study population consisted of a multistage probability sample representative of the non-institutionalized population in Catalonia, stratified by age, gender, health region (Catalonia is divided into 7 health regions according to geographical, socio-economic and demographic characteristics: Alt Pirineu-Aran; Lleida; Camp de Tarragona; Terres de l'Ebre; Catalunya Central; Girona; and Barcelona), and the size of the municipality. A total of 5598 surveys were obtained in two waves in 2015 using computer-assisted interviews administered by professional interviewers who went to the homes of interviewed subjects. In order to achieve a 100% response rate, sampling units that could not be located or declined to participate were replaced by units with the same characteristics in terms of age group, gender, municipality and nationality. In this sense, 53.2% of the surveys were first-attempt respondents, while the rest needed to be replaced at least once. Additional methodological details on the 2015 ESCA survey procedures can be found in S1 File. The current analysis includes data from 4385 surveys corresponding to people aged 15 or more years and not working in nightshift, as those groups were not required to answer the sleep-related questions in the survey.

The survey consisted of almost 500 questions about health status, health-related behaviors and use of health services, distributed in the following areas: sociodemographic variables including occupation [18] and education (n = 137); health status & health-related quality of life, including EuroQol-5D [19] (n = 44); chronic diseases (n = 42); unintentional injuries (n = 24); pharmacological treatment (n = 32); daily life limitations and disability (n = 16); preventive practices (n = 14); social support (n = 11); mental wellbeing (n = 14); dietary habits (n = 18); physical activity and mobility (n = 16); tobacco (n = 33); alcohol (n = 18); cannabis (n = 6); use of healthcare resources, including planned and unplanned hospitalizations, visits and consultations during last 15 days and last year (n = 47); material deprivation (n = 16); and, sleep habits (n = 6). Regarding sleep habits, participants were asked about sleep duration during last week: “During the last week, how many hours per night did you sleep on average?”; as well as their overall sleep health by means of the self-reported 5-item SATED scale. The scale addresses 5 positive dimensions of sleep/wakefulness that have been consistently associated with health outcomes: sleep Satisfaction (“Are you satisfied with your sleep?”); Alertness during waking hours (“Do you stay awake all day without dozing?”); Timing of sleep (“Are you asleep, or trying to sleep, between 2:00 a.m. and 4:00 a.m.?”); sleep Efficiency (“Do you spend less than 30 minutes awake at night? This includes the time it takes to fall asleep and awakenings from sleep”); and sleep Duration (“Do you sleep between 6 and 8 hours per day?”). It should be noted that while sleep satisfaction is purely subjective, each of the other questions are tied to measurable sleep/wake behaviors. Respondents indicate the frequency with which they experience or engage in each dimension, with answers ranging from 0 to 2 points (0 = “never” or “very rarely”; 1 = “sometimes”; 2 = “often" or "always”). Items on the SATED scale can be totaled to produce a single summary score, ranging from 0 (poor sleep health) to 10 (good sleep health). This is consistent with evidence indicating that sleep symptoms or problems have additive effects on health outcomes [20, 21]. SATED was created following a review of the literature on potential sleep health dimensions and their association with specific health outcomes. The specific wording of questions in the SATED scale represented an initial attempt to capture these dimensions succinctly and in a format that did not require numerical calculations by either the respondent or investigator. The ordinal responses for each item reflect increasing frequency of occurrence and are scaled in the same direction (“worst” to “best”), which permits simple summing of item scores to yield a total score. Initial development of SATED and supporting references were published in SLEEP by Buysse in 2014 [16]. Additional details on the SATED scale and its items can be found in S1 File. Self-rated health status was assessed with the question: “In general, how would you rate your health” with the possible choices being “excellent”, “very good”, “good”, “fair”, or “poor”; for the current analyses, excellent, very good and good ratings were considered as good self-rated health status. Finally, height and weight were measured using a Harpenden pocket stadiometer and a digital scale (Seca Clara 803 –Digital Scale), and BMI was calculated and categorized as follows: <18.5; ≥18.5 & <25; ≥25 & <30; ≥30; and finally, a category for subjects <18 or ≥75 years who did not have available BMI measures. Full details on the 2015 ESCA survey composition can be found in S1 File. Further methodological details of the Catalan Health Survey have been previously published [22, 23]. The complete survey, in Catalan, is publicly available from the Catalan Government web site (http://salutweb.gencat.cat/web/.content/home/el_departament/estadistiques_sanitaries/enquestes/zips/questionaris_enquesta_2015.rar [Accessed: December 21^th^, 2016]).

The ESCA survey is an official statistic of the Catalan Government. It was approved by the Consultants’ Committee of Confidential Information Management (CATIC) from the Catalan Health Department, according to the year 2000 revision of the Helsinki Declaration.

### Statistical analysis

Appropriate weighting adjustment was applied to achieve representative frequencies, as less populated territories were oversampled. Continuous variables were summarized as the mean (standard deviation (SD)), and categorical variables were summarized as percentages.

The number of chronic diseases in relation to the SATED score was assessed using non-parametric smoothing splines to represent graphically a non-linear association. The same analysis was performed according to sleep duration. The odds of having poor self-rated health status were assessed relative to different levels of each domain within the SATED instrument (low, moderate vs high), as well as sleep duration (<6h, >8h vs 6–8 h), using logistic regression models adjusted for age, sex and BMI. Socio-demographic variables, including occupation and education; lifestyle habits; and, other health-related variables were considered as potential confounders and included in the final models only if they altered the coefficient of the main variable by more than 10%. Differences in the classification accuracy of the two models were assessed by comparing the area under the receiver operating characteristic curve (AUC). Goodness of fit was assessed using the Hosmer-Lemeshow calibration test.

The main determinants of having moderate (8 ≤ SATED ≤ 9) or poor (0 ≤ SATED ≤ 7) sleep health with reference to having good sleep health (SATED = 10), were assessed using a multinomial logistic regression model. Based on bivariate analyses, independent variables were identified as candidates for the multivariate model using a p-value < 0.20 as the selection criterion. A stepwise backward strategy was used to select the final multivariate model, with a p-value < 0.05 set as the criterion for variable removal from the model. The final model was calibrated using the methodology proposed by Van Hoorde et al. [24].

R statistical software, version 3.3.1, was used for all the analyses. All tests were two tailed, and p-values < 0.05 were considered statistically significant.

### Results

A total of 4385 surveys representative of the Catalan population were included in the analyses. The main sociodemographic and health-related characteristics of the population are shown in Table 1. Briefly, 51% of the sample were women, 20% were older than 65 years of age, the mean (SD) body mass index was 25.60 (4.52) kg/m^2^, and 81.7% reported good self-rated health status. Table 2 shows the sleep habits of the population according to gender and age groups, including the mean sleep duration and SATED score and the percentage of the population reporting good sleep in each of the SATED dimensions. Overall, the population slept a mean (SD) of 7.18 (1.16) h/night and had a SATED score of 7.91 (2.17), with a possible range of 0 (poor sleep health) to 10 (good sleep health). SATED scores decreased according to age, and were lower in women. Alertness and Efficiency were the SATED dimensions most frequently indicated as impaired.

**Table 1:** Main characteristics of the population.

**Sociodemographic characteristics**	
Male gender	49%
Age (years)	47 (19)
Occupation	
I. Director/manager/university degree	20%
II. In an intermediate occupation ^a^	17%
III. Manual worker	60%
IV. Has never worked	2%
BMI ^b^	
Underweight (< 18.5)	2%
Normal weight (≥ 18.5 to < 25)	48%
Overweight (≥ 25 to < 30)	35%
Obesity (≥ 30)	15%
**Lifestyle habits**	
Tobacco use	
Daily smoker	23%
Occasional smoker	2%
Former smoker	17%
Non-smoker	58%
Alcohol ^c^	
Non-drinker	34%
Drinker (low risk)	62%
Drinker (high risk)	4%
Physical activity	
Low	26%
Moderate	59%
Vigorous	15%
**Health and health services**	
At least one chronic disease	72%
Good self-rated health status	82%
EuroQol (utility)	1 [1–1]
At least one medical visit during the last year	92%
Regular pharmacological treatment	60%
Sleeping pill use	7%

Proportion, mean (SD) or median [P25-P75], as appropriate.

^a^ Self-employed or administrative/supporting workers.

^b^ No information available for people aged < 18 years or > 74 years.

^c^ Low risk was defined as any weekly alcohol intake up to 28 standard drinks for men and 17 standard drinks for women (“standard drinks” = 10 grams of pure alcohol) in the last 12 months. Intake above that was considered high risk. Subjects without any alcohol intake in the last 12 months were considered as non-drinkers. BMI: body mass index; EuroQol: EQ-5D utility dimension.

**Table 2:** Sleep habits of the population according to age-groups and gender.

**Men**	**15–44 years**	**45–64 years**	**65–74 years**	**+75 years**
Sleep duration (hours)	7.27 ±1.02	7.00 ±1.06	7.20 ±1.27	7.58 ±1.44
SATED score	8.23 ±2.04	7.99 ±2.22	7.69 ±2.27	7.49 ±2.33
Satisfaction (%)	77	74	71	74
Alertness (%)	73	65	54	43
Timing (%)	80	79	78	75
Efficiency (%)	64	63	53	57
Duration (%)	88	81	86	84
**Women**	**15–44 years**	**45–64 years**	**65–74 years**	**+75 years**
Sleep duration (hours)	7.29 ±1.07	6.96 ±1.19	7.03 ±1.31	7.27 ±1.60
SATED score	8.17 ±2.10	7.70 ±2.31	7.20 ±2.36	7.04 ±2.59
Satisfaction (%)	73	64	60	62
Alertness (%)	75	69	52	55
Timing (%)	80	74	66	67
Efficiency (%)	62	58	50	47
Duration (%)	86	76	72	68

Mean ± SD or proportion of subjects reporting no issues, as appropriate. SATED score ranges from 0 (poor sleep health) to 10 (good sleep health). The percentage in each SATED dimension indicates the proportion of the population with the maximum score for that dimension (score = 2), that corresponds to answering “often” or “always” to the associated question.

**Fig 1:**
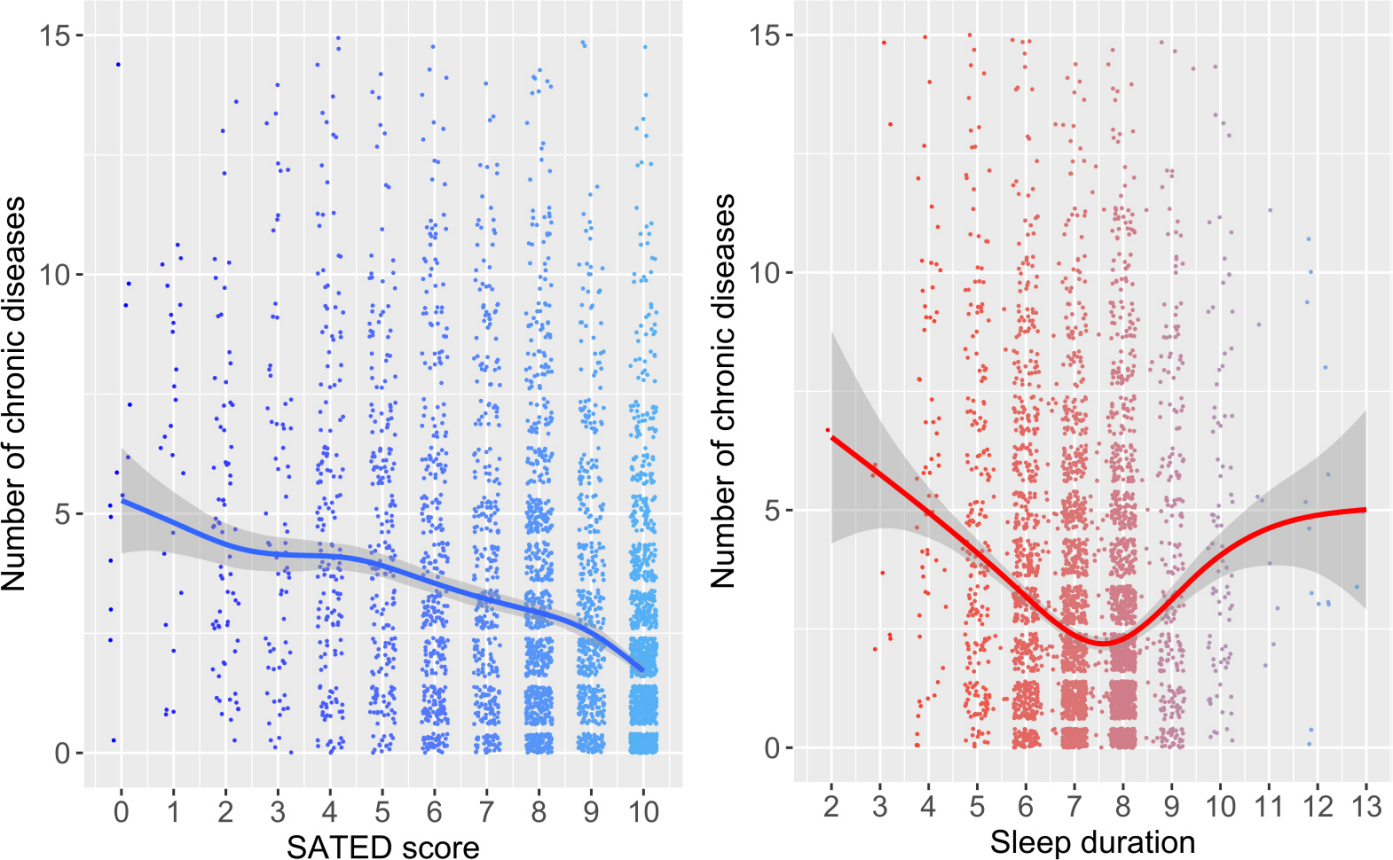
Associations of the SATED score and sleep duration with the number of reported chronic diseases. Non-parametric smoothed splines of the number of chronic diseases in relation to the SATED score and sleep duration. The gray areas correspond to 95% confidence intervals. Subjects with more than 15 reported chronic diseases are not shown but are considered in the models.

**Fig 2:**
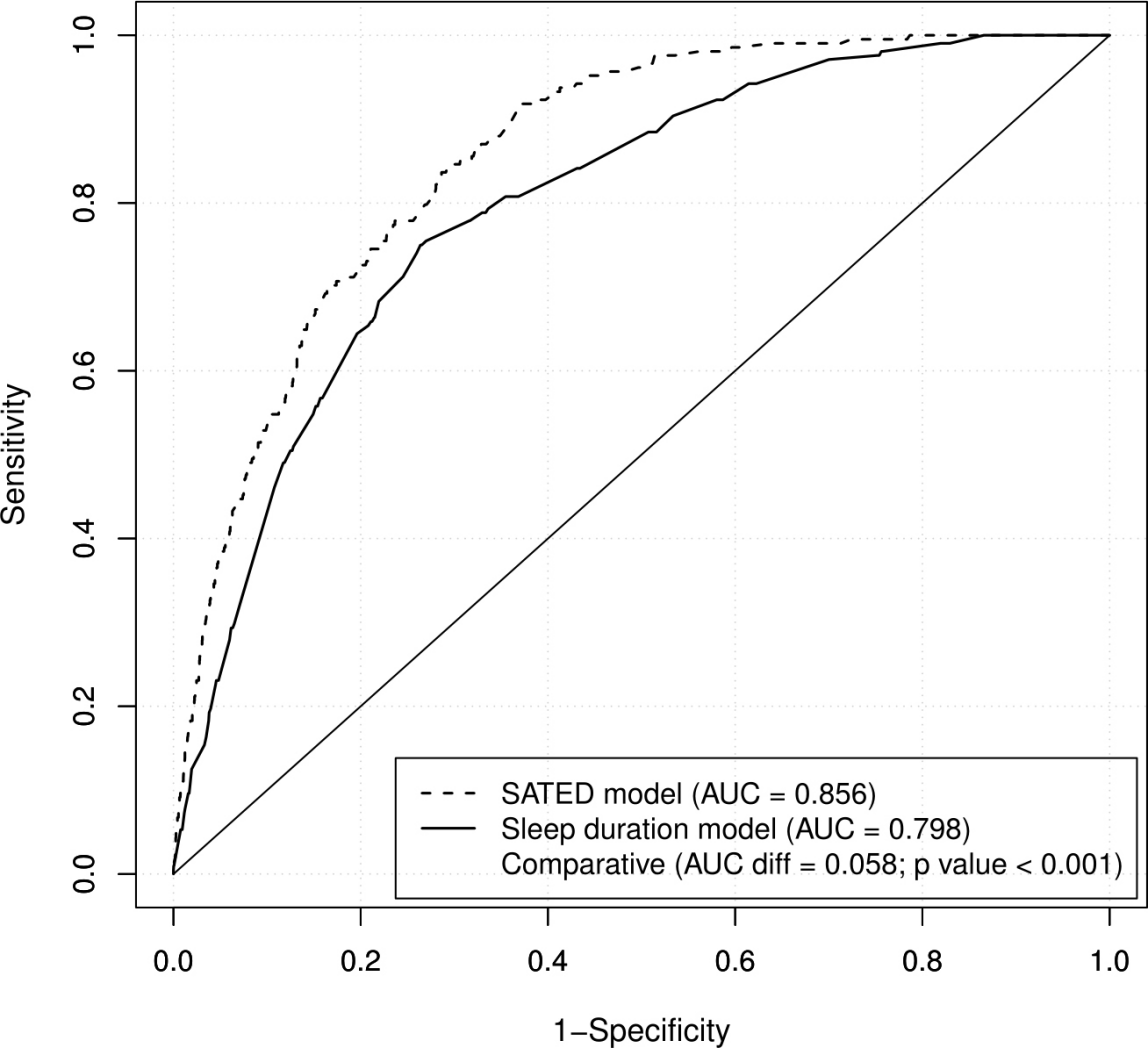
Receiver operating characteristic curves for the association between the SATED score or sleep duration and self-rated health status. Logistic regression model adjusted for age, BMI and sex. Self-rated health status assessed with the question: “In general, how would you rate your health” [“excellent”, “very good”, “good”, “fair”, or “poor”]; "fair" and "poor" were considered as poor self-rated health status. AUC: area under the curve.

Fig 1 shows the associations of the SATED scale and sleep duration with the number of reported chronic diseases. While the relationship was linear for the SATED score, a U-shaped relationship was found for sleep duration. Tables 3 and 4 show logistic regression models, adjusted for age, sex and BMI, describing the association between poor self-rated health status based on the 5 dimensions of SATED or sleep duration. Satisfaction (OR_low vs high_ (95% CI): 6.42 (3.88−9.71)) and daytime Alertness (OR_low vs high_ (95% CI): 2.71 (1.85−3.99)) were the dimensions most strongly associated with poor self-rated health status. Fig 2 shows that the SATED model performed significantly better than the sleep duration model in categorizing individuals with poor self-rated health status (AUC = 0.856 vs. 0.798; p-value < 0.001).

**Table 3:** Logistic regression models examining the association between the 5 dimensions of SATED and poor self-rated health status.

	OR	95% CI	p-value
Satisfaction: high	Ref		
Satisfaction: moderate	2.51	1.61–3.91	<0.001
Satisfaction: low	6.42	3.88–9.71	<0.001
Alertness: high	Ref		
Alertness: moderate	1.55	1.01–2.40	0.049
Alertness: low	2.71	1.85–3.99	<0.001
Timing: high	Ref		
Timing: moderate	1.11	0.73–1.66	0.627
Timing: low	1.21	0.71–2.07	0.547
Efficiency: high	Ref		
Efficiency: moderate	1.45	0.95–2.20	0.087
Efficiency: low	1.75	1.14–2.68	0.010
Duration: high	Ref		
Duration: moderate	1.10	0.69–1.77	0.662
Duration: low	0.99	0.60–1.69	0.969

Logistic regression model adjusted for age, BMI and sex. Self-rated health status assessed with the question: “In general, how would you rate your health” [“excellent”, “very good”, “good”, “fair”, or “poor”]; "fair" and "poor" were considered as poor self-rated health status.

**Table 4:** Logistic regression model examining the association between sleep duration and poor self-rated health status.

	OR	95% CI	p-value
Sleep duration = 6–8 h	Ref		
Sleep duration < 6 h	3.79	2.58–5.54	<0.001
Sleep duration > 8 h	1.87	1.16–2.99	0.009

Logistic regression model adjusted for age, BMI and sex. Self-rated health status assessed with the question: “In general, how would you rate your health” [“excellent”, “very good”, “good”, “fair”, or “poor”]; "fair" and "poor" were considered as poor self-rated health status.

Finally, Table 5 shows the main determinants of having moderate (8 ≤ SATED ≤ 9) or poor (0 ≤ SATED ≤ 7) sleep health with reference to having a good sleep health (SATED = 10). Briefly, having at least one chronic disease, low socioeconomic status and sleeping pill use were the main determinants of moderate or poor sleep health. Intermediate occupations and physical activity level were inversely associated with moderate sleep health. Likewise, quality of life (as assessed using the EuroQol) and higher educational attainment were inversely associated with poor sleep health. In contrast, being a manual worker, regular drug use and reported medical visits were associated with poor sleep health.

**Table 5:** Multinomial logistic regression model showing the main variables associated with having moderate or poor sleep health in the population.

	Moderate sleep health			Poor sleep health		
	(SATED 8–9)			(SATED 0–7)		
	OR	95% CI	p-value	OR	95% CI	p-value
Education: Secondary education	1.07	0.87–1.3	0.538	0.81	0.66–0.99	0.030
Education: University education	0.91	0.69–1.19	0.495	0.72	0.54–0.95	0.020
Occupation: II. Intermediate occupation	0.73	0.56–0.94	0.013	1	0.76–1.3	0.971
Occupation: III. Manual worker	1.1	0.88–1.37	0.402	1.29	1.01–1.64	0.039
Occupation: IV. Has never worked	0.91	0.49–1.68	0.757	1.18	0.64–2.2	0.596
Good self-rated health status	0.99	0.99–1	0.141	0.98	0.97–0.98	0.000
EuroQol (utility)	0.55	0.28–1.09	0.085	0.24	0.13–0.45	<0.001
At least one chronic disease	1.39	1.16–1.67	<0.001	1.6	1.31–1.96	<0.001
Regular pharmacological treatment	1.01	0.85–1.2	0.901	1.25	1.04–1.51	0.018
Sleeping pills	1.5	1.02–2.2	0.037	1.71	1.19–2.47	0.004
Physical activity: moderate	0.79	0.66–0.94	0.008	0.91	0.75–1.1	0.315
Physical activity: vigorous	0.81	0.62–1.04	0.1	1.11	0.84–1.46	0.459
At least one medical visit during the last year	1.28	0.99–1.64	0.057	1.47	1.1–1.97	0.010

Multinomial regression model adjusted for age, sex and BMI, with sleep health categories as the response variable. Sleep health categories: Good (SATED 10: as reference category), moderate (SATED 8−9) or poor (SATED 0−7). Odds ratios estimate the risk of moderate or poor sleep health with respect to good sleep health (SATED 10) which is the reference. EuroQol: EQ-5D utility dimension.

## Discussion

In this study, including data from a representative population sample of 4385 individuals, we assessed the sleep health of the Catalan population and the relation of this population’s sleep health to self-rated health status. A multivariate measure of self-rated sleep health decreased with age and was slightly poorer among women. Interestingly, while sleep duration increased with age, SATED scores decreased with age, thus reflecting poorer sleep health. The SATED score correlated linearly with the number of self-reported chronic diseases, while sleep duration showed a U-shaped relationship with chronic diseases. Moreover, SATED performed significantly better than sleep duration in categorizing poor self-rated health status. Among sleep health dimensions, Satisfaction and Alertness had the strongest associations with poor self-rated health status. Finally, this study identified a set of variables associated with the risk of having poor or moderate sleep health.

To our knowledge, this is the first national survey including a set of sleep questions aiming to assess overall sleep health beyond sleep duration. The sleep duration results are similar to those of other surveys in the US and Europe [25, 26]. In particular, the 2013–2014 US National Health and Nutrition Examination Survey (NHANES) reported a similar distribution of the number of hours of sleep according to age and gender [25]. Moreover, the proportion of subjects reporting having ever told a doctor about having trouble sleeping increased according to age and was higher among women [25]. This trend was inversely proportional to the SATED score trends in the current study, which decreased according to age and were always lower in women than men, regardless of age group. This gender differences could be explained by differences on the electroencephalogram’s delta activity, that could be related to subjective complaints and/or sleep fragility in the presence of environmental disturbances [27].

In our sample, Alertness and Efficiency were the sleep dimensions with the lowest ratings, although Satisfaction performed better than Efficiency in models predicting self-rated health status. Interestingly, the Satisfaction, Timing and Duration dimensions had minor differences across age groups, thus suggesting that Alertness and Efficiency are the sleep dimensions that change the most with age. Finally, while the sleep duration model predicting self-rated health status was statistically significant (Table 4), the Duration dimension in the SATED model was not (Table 3). This suggests that, although sleep duration can be a marker of overall sleep health and is associated to self-rated health status, other sleep dimensions are driving the association between sleep health and self-rated health status.

The SATED score demonstrated a significantly stronger association than self-reported sleep duration with self-rated health status. Moreover, the SATED score may provide some additional benefits: (i) it provides information on different sleep dimensions, thus allowing for public health actions focusing on specific aspects of sleep habits suitable to each population and age group, as sleep problems among young adults could affect different dimensions than in the oldest population, and (ii) it provides an overall indicator of sleep health that correlates linearly with self-rated health status and other health indicators, such as the number of chronic diseases. In contrast, sleep duration has a U-shaped relationship with most health indicators.

This study identified a set of variables that are associated with sleep health. In contrast to most published studies, which have evaluated the influence of sleep on specific diseases, our study additionally assessed the effect of different conditions on perceived sleep health, as evaluated by SATED. Chronic diseases, use of sleeping pills, and occupation were associated with moderate to poor sleep health, whereas physical activity, quality of life and higher education were associated with better sleep health. These results are in line with previous studies that described an association between several dimensions of sleep and chronic conditions such as hypertension [11], coronary disorders [5,6,28], cerebrovascular disease [29] and cognitive decline [30]. Our results are also in accordance with studies suggesting that manual occupation or lower physical activity levels are correlates of sleep disturbances [31]. Moreover, these results are in line with studies reporting an association between sleep quality or duration and health-related quality of life [14,32]. Therefore, our results provide further evidence of bidirectional influences between sleep and chronic conditions or health habits, such as physical activity.

In our study, sleep was evaluated using the SATED scale and sleep duration, which are both self-reported questionnaires. Self-report instruments are widely used to study sleep-wake function despite the existence of objective methods such as polysomnography or actigraphy. Although polysomnography is the gold standard for assessment of specific sleep disorders (e.g., sleep apnea, narcolepsy) and sleep characteristics (e.g., sleep stages, quantitative EEG characteristics), it is limited by its use of multiple sensors, typically in a laboratory environment. Similarly, actigraphy requires professionals and facilities that preclude its use in many population-based surveys. Studies assessing the correlation between objective and self-reported measures of sleep show moderate correlations, depending on the use of actigraphy [33] or polysomnography [34, 35]. However, the use of a self-reported tool in a population-based survey should be considered acceptable. The SATED scale has not yet been formally psychometrically validated, and the current manuscript does not provide such validation. Nevertheless, other scientific studies using its same 5 sleep health dimensions concluded that a multidimensional measurement of sleep health could be more useful than individual sleep measures for assessing health risks [36], as observed in our study. Our findings, demonstrating the potential utility of a multidimensional sleep health measure, provide additional impetus for formal validation studies. To date, SATED has not been used in combination with other sleep related scales and, therefore, we cannot comment on its convergent or divergent validity. However, some contrasts can be made with the general structure and intent of other widely-used instruments. The Epworth Sleepiness Scale and Insomnia Severity Index are both widely-used, but each assesses a single sleep dimension and would not be adequate for measuring the broad construct of sleep health. The Pittsburgh Sleep Quality Index covers a broader range of sleep dimensions, but it is too long and complex to be used in most population-based health surveys. The SATED scale, on the other hand, addresses multiple sleep dimensions and is both brief and easy to use. Therefore, SATED could potentially fill a gap in sleep assessment.

From a public health perspective, this study emphasizes the important association between sleep health and overall health and well-being. Moreover, framing sleep health as positive attribute highlights the importance of preventive strategies, education and health promotion initiatives aimed at improving sleep health. Sleep health could become an important component of public education and behavioral health strategies, and related interventions may be synergistic with other important health promotion activities. Finally, the study of sleep health in addition to sleep disorders, offers new and relevant research opportunities in the field of sleep medicine.

The current study has several strengths, including a large sample size, representative of the Catalan population; the use of a novel self-reported, objective method that considers several sleep dimensions; and the inclusion of a broad range of self-reported health-related variables. However, several limitations should be acknowledged: (i) the SATED scale has not yet been formally validated, although the five dimensions included on the SATED scale have been consistently associated with health outcomes, and no other validated tool is currently available to measure sleep health; (ii) the self-reported nature of SATED and other potentially relevant variables, such as the use of sleeping pills, cannot preclude some degree of reporting and social desirability biases; and, (iii) the cross-sectional design did not allow establishment of the direction of the associations.

In conclusion, our study shows that sleep health in Catalonia varies according to age and gender. Moreover, the study reveals that the SATED score had some advantages compared to self-reported sleep duration to describe sleep health, as it relates linearly to most health indicators and performs better in predicting self-rated health status. Finally, our study identifies that sleep health can be impaired or promoted by several conditions. Based on these findings, we suggest that a comprehensive assessment of sleep health, including multiple dimensions, should be considered for inclusion in national surveys, given its association with overall health status and its potential usefulness for the identification of health promotion targets.

## Supporting information

S1 File Additional methodology. (PDF)
