# Supplementary material for: Assessing sleep health in a European population: Results of the Catalan Health Survey 2015
Source: PLoS One. 2018 Apr 18;13(4):e0194495. doi: 10.1371/journal.pone.0194495 (PMC5905963; doi:10.1371/journal.pone.0194495)
Supplement: S1 File — (PDF) [file pone.0194495.s001.pdf]

S1 Supporting Information. Additional methodology

1. Summary of procedures of the Catalan Health Survey (ESCA)

ESCA is a continuous health surveying system targeting the Catalan population regardless of age. ESCA is divided into 6-month waves. In each wave a stratified random sampling by age and gender is performed in each of the 43 health-management areas (AGA) that constitute the 7 Health regions of Catalonia. The sample size in each AGA is calculated to allow for a ±5% error (this error can be slightly higher in AGA with less than 25,000 habitants). This supposes the non-proportional allocation of the quotas of the sample of each AGA, according to the population size of the AGA and the variability of the characteristics by sex and age group. Therefore, the probability of the individuals being elected is not the same. This sampling strategy ensures that after eight consecutives waves (≈20,000 surveys) the surveys in a given AGA are representative of the AGA population. On the other hand, a single wave (≈2,500 surveys) is required to achieve representativeness at the Catalan level with a ±2% error margin, but a weighting system needs to be applied to compensate the over-sampling of less populated AGA. This methodology guarantees that, after the application of weights, the surveys in a given wave are representative of the Catalan population with a ±2% error margin [1].

The Catalan Institute of Statistics (Idescat) elaborates the list of titular and substitute sample units with all the logistic data need for interviewers to go to their dwellings to administer the surveys. To begin with, the list includes 5 substitutes per titular sample unit, but up to 5 additional substitutes can be provided if needed. All titular sample units receive an informative letter two week before the interview. At the time of the interview, professional interviewers go to the dwelling of each sample unit with the support of a computer. In case of impossibility to interview the selected subject after a given number of attempts, interviewers proceeded to the dwelling of the 1st substitute sample unit, personally handing the informative letter at the time of the interview. This procedure is repeated until a 100% response rate is achieved.

All ESCA interviews are delivered by professional interviewers with adequate formation, following an Interviewer's Protocol including detailed information on how the interviewer must present himself/herself; the attitude towards the interviewed person; how to contact the selected subjects; the procedure to follow in case of substitutions; the registration of incidents; and finally, how to ask the questions of each questionnaire included in the ESCA survey. The duration of the interviews depends on the composition of the survey of each ESCA waive,

ranging from 45 to 90 minutes. Codification and depuration of all collected data is done by qualified personnel of the Catalan Health Department.

2. Composition of the 2015 ESCA survey

Different versions of the ESCA survey are elaborated in each 6-month wave so they can adapt to the surveyed subject characteristics (adults, children under 15 years-old, and caregivers of subjects that cannot respond the survey due to disease or incapability). All measurement instruments used in ESCA are validated at the national and international levels and are used without further modifications, with the exception of experimental questions added to specific waves of the ESCA survey, such as the sleep-related questions incorporated to ESCA 2015. Additionally, it must be noted that not all sections of the main survey are asked to every participant (i.e. gender-specific questions).

The main components of ESCA 2015 and corresponding references are listed below:

Health-related quality of life (EQ-5D-5L): (i) Euroqol Group. EuroQol-A new facility for the measurement of healthy-related quality of life. Health Policy, 1990;16(3):199-208; (ii) Rabin R, de Charro F. EQ-5D: a measure of health status from the EuroQol Group. Ann Med 2001;33(5):337-43.

Chronic conditions; limitations, disabilities and personal autonomy; and, material deprivation: (i) Minimum European Health Module (MEHM). Eurostat. European Health Interview Survey (EHIS wave 2) Methodological manual. 2013 edition; and, (ii) Buratta V, Forva L, Gargiulo L, Gianicolo E, Prati S, Quattrociocchi L. Development of a common instrument for chronic physical conditions. A EUROHIS: Developing Common Instruments for Health Surveys. Cap 3, 21-34. IOS Press, 2003.

Occupation*: Domingo-Salvany A, Bacigalupe A, Carrasco JM, Espelt A, Ferrando J, Borrell C. Propuestas de clase social neoweberiana y neomarxista a partir de la Clasificación Nacional de Ocupaciones 2011. Gac Sanit. 2013; 27(3): 263-72.

Mental well-being (WEMWBS): Castellví P, Forero CG, Codony M, Vilagut G, Brugulat P, Medina A et al. The Spanish version of the Warwick-Edinburgh mental well-being scale (WEMWBS) is valid for use in the general population. Qual Life Res.2014;23(3):857-68.

Sleep health (SATED): Buysse DJ. Sleep Health: Can We Define It? Does It Matter? SLEEP 2014;37(1):9-17.

Physical Activity (IPAQ): (i) Craig CL, Marshall AL, Sjöström M, Bauman AE, Booth ML, Ainsworth BE et al. International physical activity questionnaire (IPAQ): 12-country reliability and validity. Med Sci Sports Exerc. 2003;35(8):1381-95; (ii) Hallal PC, Victoria CG. Reliability and validity of the International Physical Activity Questionnaire (IPAQ). Med Sci Sports Exerc. 2004;36(3):556.

Mediterranean diet adherence (PREDIMED): (i) Martínez-González MA, García-Arellano A, Toledo E, Salas-Salvadó J, Buil-Cosiales P et al. A 14-Item Mediterranean Diet Assessment Tool and Obesity Indexes among High-Risk Subjects: The PREDIMED Trial. PLoS ONE. 2012;7(8):e43134; (ii) Schröder H, Fitó M, Estruch R, Martínez-González MA, Corella D, Salas-Salvadó J et al. A short screener is valid for assessing Mediterranean diet adherence among older Spanish men and women. J Nutr. 2011;141(6):1140-5.

Alcohol intake: Rodríguez-Martos A, Gual A, Llopis JJ. La “unidad de bebida estándar” como registro simplificado del consumo de bebidas alcohólicas y su determinación en España. Med Clin (Barc) 1999;112(12):446-50.

* ESCA uses as a social class proxy the proposal of occupational social class defined by the Working Group on Social Determinants of the Spanish Society of Epidemiology. This classification consists of 3 main categories: (i) directors, managers and professionals with a university degree; (ii) intermediate occupations: self-employed or administrative/supporting workers; (iii) manual workers. In order to classify a given subject into one of the above-mentioned categories, the current or former occupation of the most qualified subjects in the dwelling is considered. If none of the subjects living in the dwelling has ever worked a “has never worked” category is used. Additionally, ESCA has questions on educational status. No questions on income areavailable.

3. SATED scale

The SATED –Satisfaction, Alertness, Timing, Efficiency and Duration– scale is a tool for the measurement of sleep health proposed by Buysse in 2014 [2]. SATED is the result of a comprehensive review of the literature on potential sleep health dimensions and their

association with specific health outcomes in an attempt to create a tool capable of quantifying sleep health. During the development of the SATED tool, dimensions were considered as appropriate indicators of sleep health if they met the following criteria: (i) each dimension was associated with health outcomes, albeit with somewhat different outcomes for each dimension; (ii) they could each be expressed in positive terms; (iii) dimensions should be measurable across self-report, behavioral, and physiological levels of analysis; and (iv) each dimension had good face validity or ecological validity. The five dimensions of sleep that resulted the most relevant were: sleep duration (the total amount of sleep obtained per 24 hours); sleep continuity (the ease of falling asleep and returning to sleep); timing (the placement of sleep within the 24-hour day); alertness/sleepiness (the ability to maintain attentive wakefulness); and, satisfaction/quality (the subjective assessment of having "good" or "poor" sleep). In order to develop the shortest questionnaire targeting the five identified dimensions, five questions were developed using existing literature to identify the thresholds that discriminated the most the associations between the proposed sleep dimensions and health risks. The proposed questions were: sleep Satisfaction ("Are you satisfied with your sleep?"); Alertness during waking hours ("Do you stay awake all day without dozing?"); Timing of sleep ("Are you asleep, or trying to sleep, between 2:00 a.m. and 4:00 a.m.?"); sleep Efficiency ("Do you spend less than 30 minutes awake at night? This includes the time it takes to fall asleep and awakenings from sleep"); and sleep Duration ("Do you sleep between 6 and 8 hours per day?"). It should be noted that while sleep satisfaction is a purely subjective question, each of the other questions are tied to measurable sleep/wake behaviors. Respondents indicate the frequency with which they experience or engage in each dimension, with answers ranging from 0 to 2 points (0 = "never" or "very rarely"; 1 = "sometimes"; 2 = "often" or "always"). Items on the SATED scale can be totaled to produce a single summary score, ranging from 0 (poor sleep health) to 10 (good sleep health). This is consistent with evidence indicating that sleep symptoms or problems have additive effects on health outcomes [3,4].

Several limitations regarding the SATED scale must be acknowledged. First, the SATED scale has not yet been formally psychometrically validated. Although it was developed with an a priori conceptual model based upon previous empirical observations, the unique contribution of the selected domains has yet to be demonstrated. Similarly, the weight of each of the domains to the overall sleep health may not be proportional. In this sense, an ongoing validation project for SATED will provide the final sleep dimensions, questions, thresholds and weights if needed.

Therefore, the preliminary version of SATED used in the current analysis may vary from the to-be-published validated SATED questionnaire. In the meanwhile, the only sources supporting the value of SATED are scientific initiatives using the same sleep health dimensions. For instance, a 2017 study from Furihata using the SATED dimensions concluded that both individual sleep dimensions and the aggregate measure of sleep health were associated with prevalent depression and the longitudinal development of clinically significant depression symptoms [5]. These results suggest that the specific dimensions, cut-off values and categorizations could reflect sleep health, and thus be useful. Moreover, it points out that individual dimensions occur in conjunction with the others and that a multivariate measurement could be more useful than individual measures for assessing health risks related to sleep health.

References

1. Alcañiz-Zanón M, Mompart-Penina A, Guillén-Estany M, Medina-Bustos A, Aragay-Barbany JM, Brugulat-Guiteras P, Tresserras-Gaju R. New design of the Health Survey of Catalonia (Spain, 2010-2014): A step forward in health planning and evaluation. Gac Sanit 2014; 28(4): 338-340. [Article in Spanish]

2. Buysse DJ. Sleep health: can we define it? Does it matter? SLEEP 2014; 37 (1): 9-17.

3. Laugsand LE, Vatten LJ, Platou C, Janszky I. Insomnia and the risk of acute myocardial infarction: a population study. Circulation. 2011; 124 (19): 2073-81.

4. Vgontzas AN, Liao D, Bixler EO, Chrousos GP, Vela-Bueno A. Insomnia with objective short sleep duration is associated with a high risk for hypertension. SLEEP 2009; 32 (4): 491-7.

5. Furihata R, Hall MH, Stone KL, Ancoli-Israel S, Smagula SF, Cauley JA, Kaneita Y, Uchiyama M, Buysse DJ; Study of Osteoporotic Fractures (SOF) Research Group. An Aggregate Measure of Sleep Health Is Associated With Prevalent And Incident Clinically Significant Depression Symptoms Among Community-Dwelling Older Women. Sleep 2017; 40(3).
